# Bilingualism is always cognitively advantageous, but this doesn’t mean what you think it means

**DOI:** 10.3389/fpsyg.2022.867166

**Published:** 2022-08-16

**Authors:** Guilherme Sanches de Oliveira, Maggie Bullock Oliveira

**Affiliations:** ^1^Biological Psychology and Neuroergonomics, Technische Universität Berlin, Berlin, Germany; ^2^Faculty of Social and Cultural Sciences, European University Viadrina, Frankfurt Oder, Germany

**Keywords:** bilingualism, multilingualism, language ideologies, sociolinguistics, communities of practice, information processing, embodied cognition, ecological psychology

## Abstract

For decades now a research question has firmly established itself as a staple of psychological and neuroscientific investigations on language, namely the question of whether and how bilingualism is cognitively beneficial, detrimental or neutral. As more and more studies appear every year, it seems as though the research question itself is firmly grounded and can be answered if only we use the right experimental manipulations and subject the data to the right analysis methods and interpretive lens. In this paper we propose that, rather than merely improving prior methods in the pursuit of evidence in one direction or another, we would do well to carefully consider whether the research question itself is as firmly grounded as it might appear to be. We identify two bodies of research that suggest the research question to be highly problematic. In particular, drawing from work in sociolinguistics and in embodied cognitive science, we argue that the research question of whether bilingualism is cognitively advantageous or not is based on problematic assumptions about language and cognition. Once these assumptions are addressed head on, a straightforward answer to the question arises, but the question itself comes to seem to be a poor starting point for research. After examining why this is so, we conclude by exploring some implications for future research.

## Introduction

Is the ability to speak more than one language cognitively beneficial, cognitively detrimental, or cognitively neutral? In the past couple of decades the literature seems to have converged on a mixed conclusion: bilingualism confers to the speaker some cognitive advantages while also bringing with it some cognitive disadvantages.

On the one hand, for instance, there is a wealth of evidence suggesting that bilinguals exhibit increased executive function and executive control, including better performance than monolinguals in some problem-solving tasks, especially those requiring self-monitoring and the inhibition of irrelevant information (Bialystok et al., 2008; Festman et al., 2010; Pelham and Abrams, 2014). This positive relation between bilingualism and greater executive function has been found to apply throughout the lifespan, from childhood to old age (Bialystok, 2007). Yet it’s especially later in life that the advantage appears to be greater, as bilingualism is associated with increased cognitive flexibility and mental health benefits for the elderly, including delayed dementia onset (Fox et al., 2019).

On the other hand, however, many studies (including some of the same ones already cited) also report clearly negative cognitive effects of bilingualism. Most prominent among these cognitive disadvantages are a deficit in lexical access and retrieval (Bialystok et al., 2008; Pelham and Abrams, 2014) and worse performance in speech production tasks (Sadat et al., 2012), as well as diminished metacognitive efficiency (Folke et al., 2016). Not only that, but even some of the cognitive advantages cited above have come to be questioned in the recent literature. For instance, in a meta-analysis Lehtonen and colleagues propose that the findings showing an advantage of bilingualism with regard to executive function suffer from publication bias, and they conclude that, correcting for this bias, the cognitive advantage is minimal if at all existent: “If some enhancement of cognitive control functions exists attributable to bilingualism, it is restricted to very specific circumstances, and its magnitude and extent are modest” (Lehtonen et al., 2018, p. 416). Negative results like these are made even more impactful in light of research that more generally challenges psychometric constructs such as “inhibition” (Rey-Mermet et al., 2018).

Despite this recent flood of work arguing in favor of these diverse answers, the debate is far from new. Writing in 1966, Diebold (1968) notes that, among educators in the United States, the dominant view at that time was that bilingualism is “a damaging experience for the child, one which poses hurdles to the child’s intellectual development and later emotional adjustment” (pp. 1–2). And even while he had reservations about extreme versions of this view, Diebold also cites prior research, from the 1950s and 60s, to suggest that at that point the idea that bilingualism is cognitively deleterious was scientifically well founded: “Let this be clear from the start: competent recent surveys of the literature (…) do reveal that there is an association between bilingualism and lower intelligence ratings” (p. 2). In reality, however, then as now, evidence could be found supporting different conclusions about the cognitive advantages or disadvantages of bilingualism. Doctoral dissertations from that period make this point very clear.

Consider, for instance, Potts’s (1965) doctoral work on the effect that one year of instruction in a foreign language (French) played in the reading proficiency and overall school achievements of monolingual American first grade students. The usual recommendation then was that second-language instruction should be provided only later, after students had developed strong reading and writing skills in English, to avoid interference from the foreign language. But having found no cognitive effect, whether positive or negative, Potts (1965) proposed that first grade was a perfectly fine time to include second-language instruction in the curriculum. For another example, in contrast with Potts’s focus on monolingual American first-graders who were starting to learn a foreign language at school, Anisfeld’s (1964) doctoral research studied teenagers and adults with life-long experiences with two languages. Anisfeld defined cognitive functioning in terms of performance in intelligence tests and related tasks, and found that, controlling for IQ scores, subjects who were proficient in more than one language had a clear cognitive advantage: “bilinguals are superior to monolinguals on intellectual tasks requiring abilities to abstract rules and manipulate symbols and to maintain a flexible approach or a flexibility set to problem solving” (1964, p. 87).

On the surface level, early studies like these show that the co-existence of evidence both in support of and against claims of cognitive advantages to bilingualism is not a new phenomenon. More fundamentally, however, these studies show that the research question itself has a relatively long history (i.e., long for psychology, neuroscience, and allied fields), and that more than sixty years ago it was already seen as an important frontier in research. This long history gives the research question an aura of credibility, which motivates new work to focus on how to improve prior methods so as to more conclusively answer the question and determine whether bilingualism is cognitively advantageous or cognitively disadvantageous in certain respects or others. But having a long history does not mean that the research question is in fact a good one.

In contrast with contributions trying to answer the research question in one direction or another, our goal in this paper is to examine the research question itself. We think that the research question is problematic for a number of different reasons. Here we focus on just two types of reasons stemming from work in sociolinguistics and in embodied cognitive science. As we propose, the question of whether bilingualism is cognitively advantageous or cognitively disadvantageous, as currently framed, is built upon inadequate essentialist and internalist assumptions about the nature of language(s) and about the nature of cognition. We examine these assumptions in two separate sections, one titled “What is ‘bilingualism’ such that it may be cognitively advantageous or disadvantageous?” and the other titled “What is ‘cognition’ such that something may be cognitively advantageous or disadvantageous?” We conclude in the final section by articulating how these different perspectives on language and on cognition motivate skepticism about *any* of the usual answers to the research question. Ultimately, we conclude that linguistic knowledge is always cognitively beneficial—but this does not mean what most people would think it means: rather than answering the research question, these ideas from sociolinguistics and embodied cognitive science suggest that the research question is misguided and not as solid a starting point for research as it might have seemed.

## What is “bilingualism” such that it may be cognitively advantageous or disadvantageous?

In this section we will present a number of different but related reasons for seeing “bilingualism” as a problematic category. Each point will build upon the previous one, but as we continue moving through them, we come to a more nuanced appreciation of the inadequacy of the conceptual framework that grounds the research question of bilingualism’s cognitive advantage or disadvantage. As will become clear, the research question is not a good starting point for research because the concepts “bilingual” and “bilingualism” are not clear enough to make it possible to answer the question.

### “Bilingualism” is problematic because it’s in continuity with “monolingualism”

**Figure 1:**
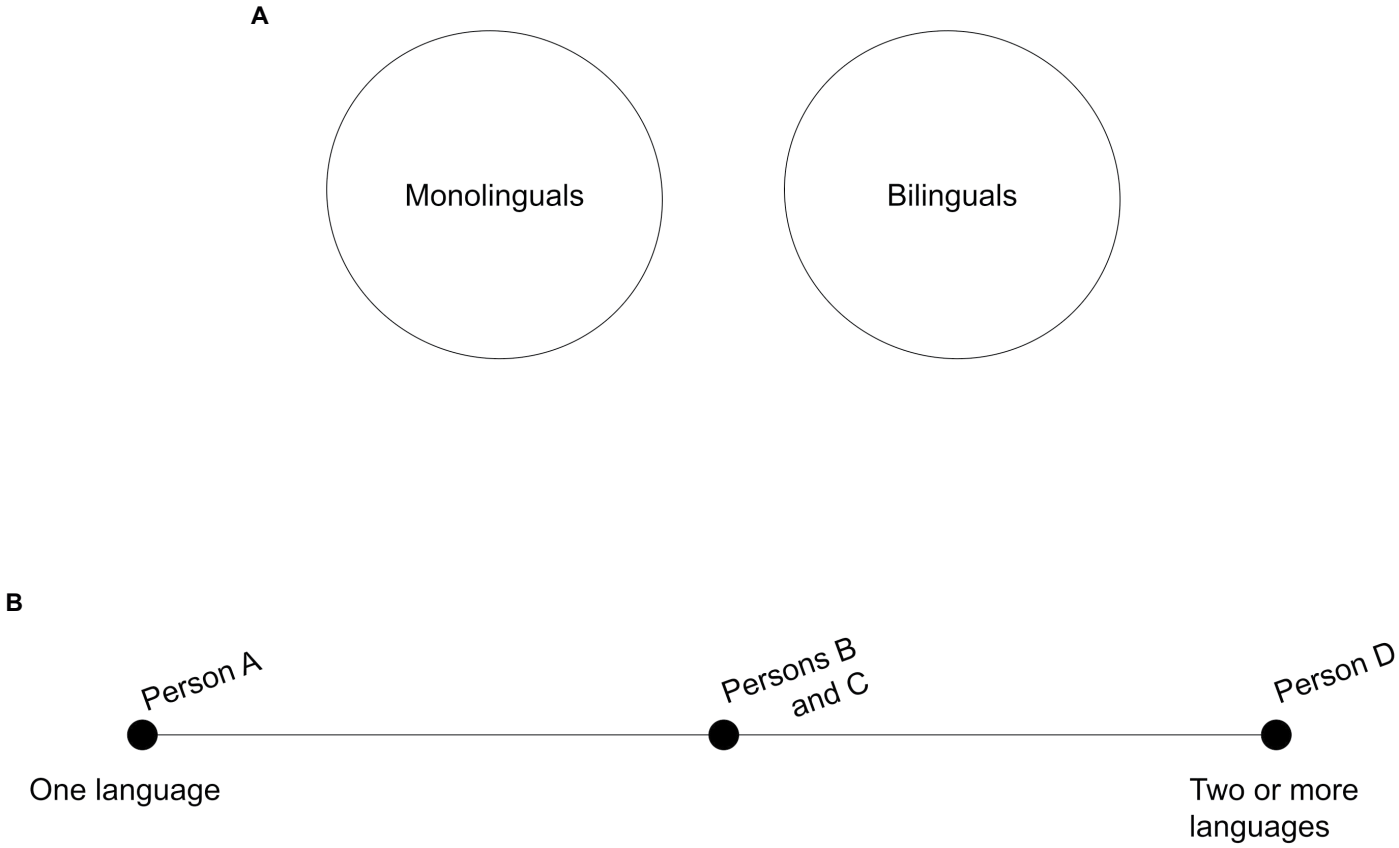
Conceived of as mutually exclusive categories, monolingualism and bilingualism divide all people into two groups based on whether they can speak one language or more than one (top, **A**). But there are many people who seem to fall somewhere in the middle, suggesting that it is better to understand bilingualism and monolingualism as in continuity with each other rather than as discrete categories (bottom, **B**): here, person A is monolingual, and person D is bilingual, but persons B and C have partial knowledge of more than one language and cannot be properly categorized at either extreme of the continuum.

It might seem intuitive to think that monolingualism and bilingualism are discrete, mutually exclusive categories: either you know only one language and are therefore monolingual, or you know more than one language and are therefore bilingual. But this assumption is clearly inadequate, and this is not news (see Surrain and Luk, 2019 for a review of inconsistent definitions used by researchers to distinguish between bilinguals and monolinguals). Even the earliest scientific research on bilinguals recognized the need for a more nuanced conceptualization of people’s language knowledge. Instead of seeing monolingualism and bilingualism as distinct “boxes” with no overlap, it makes more sense, as Anisfeld (1964) proposed, to understand them as a continuum. In this view, although there are people at both extremes—i.e., people who are unquestionably monolingual and others who are unquestionably bilingual—there are many others who fall somewhere in between and who have partial knowledge of additional languages. This gradation of linguistic knowledge makes the label “bilingual” not very informative if defined in complete opposition to “monolingual”: how much of another language do you need to know in order to be promoted from one box (“monolingual”) to the other (“bilingual”)? Is a little bit of knowledge sufficient, and you are bilingual even if not fully proficient? Or is full proficiency a prerequisite for you to count as bilingual? Understanding the categories as in continuity with one another makes it possible to recognize that people’s varying levels of knowledge count in favor of seeing them as falling somewhere along the bilingualism spectrum (see Figure 1). A first reason why “bilingualism” is a problematic concept, then, is that it’s not a discrete category completely distinct from monolingualism: although it might seem intuitive, the assumption of a dichotomy oversimplifies the realities of language knowledge and learning, which is a pitfall that recent research has been careful to avoid (see, e.g., Gullifer and Titone, 2020, 2021a; Bialystok, 2021; Kremin and Byers-Heinlein, 2021; Tiv et al., 2021).

### “Bilingualism” is problematic because of inter- and intra-individual variation in language skills

Moving to a conception of monolingualism and bilingualism as a continuum is an improvement from the dichotomous conception, but it is still inadequate. Just because someone can speak well in two or more languages, it does not follow that they can write well in all of those languages, and vice versa. Thinking in terms of a single, absolute continuum that applies to everyone (i.e., a continuum in which different individuals can be placed and compared to each other) fails to account for this variation *between* individuals, as well as *within* a single individual, across different *skills* (see, e.g., Wagner et al., 2022). In the recent literature, there have been proposals to combine different continua for separate variables (e.g., Kremin and Byers-Heinlein, 2021): while efforts like these improve our ability to identify complex *inter*-individual differences, they still fall short from fully capturing the multifaceted nature of bilingualism as exhibited in *intra*-individual variation in skills.

To refer once again to Figure 1, consider how the idea of an absolute, objective continuum makes it impossible to acknowledge the complexity of person B’s knowledge. Person B can be distinguished from person D in having only partial overall knowledge of more than one language, but the absolute impersonal continuum does not tell us anything beyond that. It could be that some of B’s skills (e.g., reading) are nearly equivalent to those of a fully proficient bilingual such as person D, even if other skills fall short. *There’s no “language ability in general”* but only ability in different language skills, and these skills do not all develop together and at the same pace.

**Figure 2:**
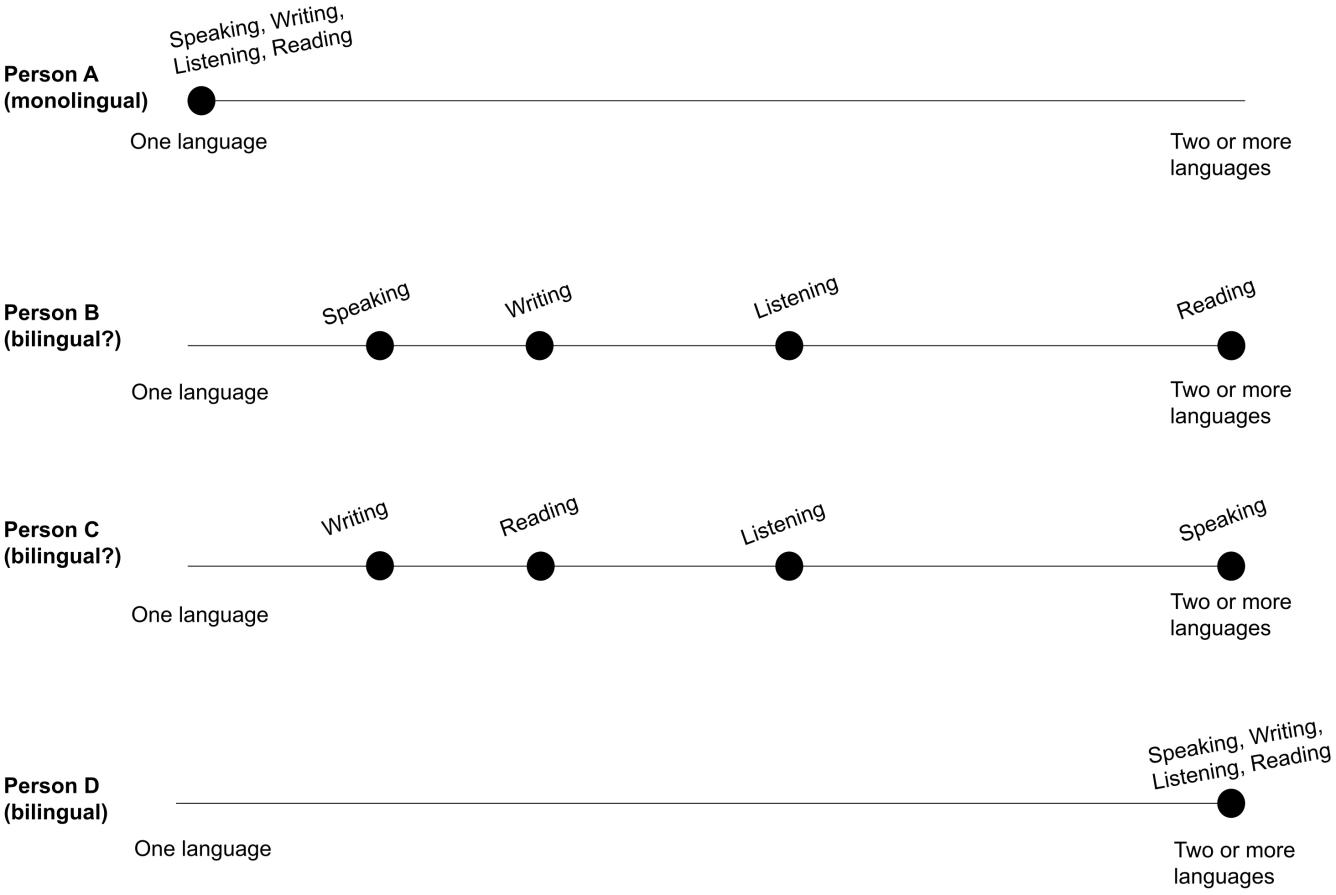
An improvement on Figure 1B is to think not of a single absolute continuum that applies to all people, but to acknowledge that each individual will have a continuum of skills they can employ in one language (left side) or in multiple languages (right side), or somewhere in the middle. As a monolingual, person A’s skills are all on the extreme left side of the continuum. Similarly, as a fully proficient bilingual, person D’s skills are all on the extreme right side of the continuum. Persons B and C have partial proficiency in more than one language but they differ from one another with respect to how advanced each of their skills is in more than one language. For instance, person B is fully proficient in speaking in one language but is very limited in other languages, while being able to read highly proficiently in more than one language; contrast this distribution with person C’s.

In contrast with a continuum between monolingualism and bilingualism that is absolute and impersonal, one that applies to everyone at once, it seems better to think in terms of individuals having their own continuum in which their monolingual and bilingual skills stand (see Figure 2). This idea is present at the foundations of virtually all formal language instruction and it’s also something that standardized tests capture well. The language abilities of individuals aren’t monolithic blocks of homogeneous linguistic knowledge, but instead vary across different receptive skills (listening and reading) and productive skills (speaking and writing). This has long been understood in second language acquisition and teaching (see, e.g., Davies, 1976), and the same insight continues to guide sociolinguistic research, where continua are used to illustrate an individual’s abilities across different languages and skills (see, e.g., Blommaert and Backus, 2013). Conceptualizing the monolingualism-bilingualism continuum in terms of the particular skills of individuals makes it possible to qualify the comparison between persons B and D in a way that the models shown in Figure 1 did not allow. Moreover, it makes it possible to acknowledge that different people who are not fully proficient bilinguals can have different bilingual language abilities, as Figure 2 illustrates with the similarities and differences between persons B and C.

This well-known way of thinking about language ability reveals a second reason why we think “bilingualism” is a problematic concept. Acknowledging that monolingualism and bilingualism are in continuity with each other is a step in the right direction, but it’s not enough because it neglects the ways in which bilinguals can differ from one another in their skills. On its own the label “bilingual” is just not very informative (Surrain and Luk, 2019), which is why researchers increasingly find it necessary to take into account the dynamic nature of language ability and the diversity, across a wide range of variables, between individuals who might otherwise have appeared to be comparable as “bilinguals” (see, e.g., Hartanto and Yang, 2016, 2020; Gullifer et al., 2018; Gullifer and Titone, 2020; Sulpizio et al., 2020; Kremin and Byers-Heinlein, 2021). Given the complex differences in skill that people can have, it’s not clear what the category “bilingual” should include and what it should leave out. Are B and C bilingual? Compared to A, the answer seems to be obviously affirmative. But what about compared to person D?

### “Bilingualism” is problematic because language and language skills are context specific

In order to more accurately describe people’s linguistic abilities, the move from an objective, absolute continuum that applies to all individuals toward individualized distributions of productive and receptive skills is an improvement. But it’s still not quite so good because, even if well intended, it can lead to thinking of language ability as a set of decontextualized skills.

Recent work in sociolinguistics provides reasons to see talk of skills as too simplistic. Blommaert and Backus (2013) criticize the way standardized proficiency tests focus on skills. In their example, they consider how one bilingual person’s abilities would be assessed by the widely used testing scale of the Common European Framework of Reference for Languages. They explain:

If we apply the Common European Framework levels for language proficiency, our subject would undoubtedly score a C2—the most advanced level of proficiency—for English, when the language test concentrates on academic genres of text and talk. The same subject, however, would score A2—the most elementary level of proficiency—if the test were based on how he would interact with a medical doctor, a plumber, an IT helpdesk operative, an insurance broker, and so on. So, ‘how good is his English’ then? Let it be clear that this question can only be appropriately answered with another one: ‘which English?’ (Blommaert and Backus, 2013, p. 30).

In the previous sub-section we suggested that it’s not appropriate to think of “language ability in general” but rather in terms of ability levels in specific skills. However, these authors show that even this is not good enough because even skills are context dependent: there’s no “speaking in general” but speaking in this kind of context, that other kind of context, and so on. From this it follows that proficiency tests are necessarily limited: skills are grounded in particular activities, and any given test can only simulate a limited range of activities (Shardakova, 2022).

This criticism of general language skills echoes broader interest in language instruction for specific purposes. The assumption behind programs offering “language for specific purposes” is that students are best served when the language they learn is tailored to the particular activities and contexts they wish to engage in. In English for Specific Purposes, for example, specific types of English contexts include academic English, business English, and even more specifically, civil engineering English (Otto, 2021), brewing English (Orsi and Orsi, 2002), hospitality and tourism English (Hsu, 2014), for just a few examples. The purpose-specificity of linguistic skill has important implications not only for instruction, but also for proficiency testing (see Grapin, 2017 for a helpful historical overview).

Similar to the critiques of generic language skills from sociolinguistics and language teaching is the related emphasis other literatures have placed on recognizing that language is made up of particular ways of speaking and writing according to context. Illustrating this concern, some researchers have turned to investigating “registers,” which they describe as “any language variety defined by its situational characteristics, including the speaker’s purpose, the relationship between speaker and hearer, and the production circumstances” (Biber, 2009, p. 823; see also Biber and Conrad, 2019, Bowcher, 2019, Szmrecsanyi, 2019). This body of research highlights the fact that language ability cannot be properly understood other than in relation to specific situations of use. And along similar lines, researchers in psycholinguistics have pushed for taking into account multiple linguistic variables to paint a richer picture of “bilinguals” in terms of their potentially very diverse experiences and exposure to languages (see, e.g., Hartanto and Yang, 2016; Gullifer et al., 2018; DeLuca et al., 2019, 2020; Gullifer and Titone, 2020, 2021a).

**Figure 3:**
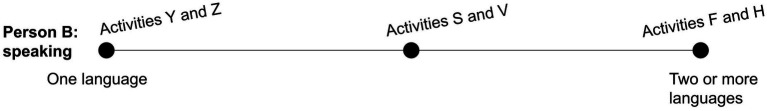
An illustration of how a person’s speaking proficiency is differently distributed across all of the activities this person engages in in life (e.g., communicating with coworkers, bargaining at a flea market, participating in a religious ceremony). Some or all of the different canonical language skills may be at play in each of these activities. The continuum displayed in this figure represents how proficiency in a single skill (here, speaking) can be distributed across different activities for the same person. Person B’s speaking abilities in activities Y and Z might be limited to one language (e.g., they can do Y only in English, and Z only in French), whereas in activities F and H they can speak fluently in two or more languages. It could be that, for this specific person, if they could engage in activity Y (say, contacting their landlord) in writing rather than orally, their ability to succeed in that situation would be greatly improved.

In light of these considerations, it becomes clear that language skills such as writing and speaking cannot be properly accounted for in a vacuum, apart from the many different situations, contexts, and activities in which individuals write and speak, for example. Recognizing that an individual’s language knowledge is distributed along a continuum of skills (as shown previously in Figure 2) is good, but does not go far enough. Language skills are not context independent: there is no “writing in general” or “speaking in general,” but each of these competencies are inextricable from some context-specific, real-life activity or other in which the person is more or less well equipped to succeed. To better capture the complex reality of language knowledge, it’s more appropriate to characterize individuals not in terms of a continuum of proficiency in general skills but a continuum *within* specific skills (e.g., speaking) distributed along specific activities in which the person is capable of successful engagement in one or more languages (see Figure 3). Instead of thinking of someone’s “speaking in general” as being more or less advanced, different lines of research like the ones mentioned above increasingly recognize that a person’s speaking may be at an advanced level for some types of activities but at a lower level for other types of activities, and that the same applies to other canonical skills.

This being the case, the category “bilingual” is problematic because, on its own, it fails to acknowledge (i) the many ways in which language itself varies in different contexts and types of activities, and accordingly, (ii) the many ways in which individual language ability is always specific to some contexts and types of activities and not others, varying even within a single skill (e.g., speaking). Not only do bilinguals differ from one another in how their skills are distributed across levels of proficiency, but their level of proficiency even in a single skill will vary according to specific contexts and types of activities (Blommaert and Backus, 2013). In light of this, many psycholinguistic researchers increasingly consider how individual differences in language abilities arise from distinct situational contexts of language use as well as different, changing life experiences (see, e.g., Gullifer et al., 2018; Gullifer and Titone, 2020; Tiv et al., 2021). Acknowledging this complexity of variation adds greater granularity to the question “What counts as being bilingual?,” and we cannot answer the research question about bilingualism’s cognitive advantages and disadvantages without first answering this one.

### “Bilingualism” is problematic because “full proficiency” is problematic

So far we have considered different and increasingly nuanced reasons why “bilingual” and “bilingualism” are seen as problematic categories. Figures 2 and 3 improved upon the view of an absolute, objective continuum that applies to everyone (Figure 1B) by recognizing the intra-individual variability of linguistic skills (Figure 2), and even better, the diversity of activities in which the individual skills of individual people are grounded (Figure 3). As shown in Figure 3, context-specific abilities range from fully proficient in one language to fully proficient in more than one language. However, if language ability is more adequately understood in terms of the proficiency an individual has developed *for engaging in specific activities*, it becomes crucial to think more carefully about what we mean by “proficiency” and, in particular, about *which activities* matter and which do not for considering an individual as a fully proficient bilingual.

It can be tempting to think that having full bilingual proficiency means to be like a monolingual in each of the languages you speak. Pennycook and Makoni (2020) give this view of bilingualism the name “plural monolingualisms” because it assumes that a plurality of monolingualisms can coexist inside a single person. Their critique resonates with Grosjean (1985), who problematized the idea that bilinguals need to have the sum of two complete monolingual repertoires and instead emphasized that each bilingual person has a unique linguistic profile. But we think it’s important to extend this critique to monolinguals as well. In the progression from Figure 2 to Figure 3 we highlighted that each bilingual person’s language skills are context specific. The same point applies to so-called monolinguals: the skills of each “monolingual” individual are context dependent, and no monolingual develops the ability to engage equally successfully in all of the contexts that are possible in their society. As Blommaert (2010) puts it, “No one knows all of a language. That counts for our so-called mother tongues and, of course, also for the other ‘languages’ we acquire in our life time. Native speakers are not perfect speakers” (p. 103).

**Figure 4:**
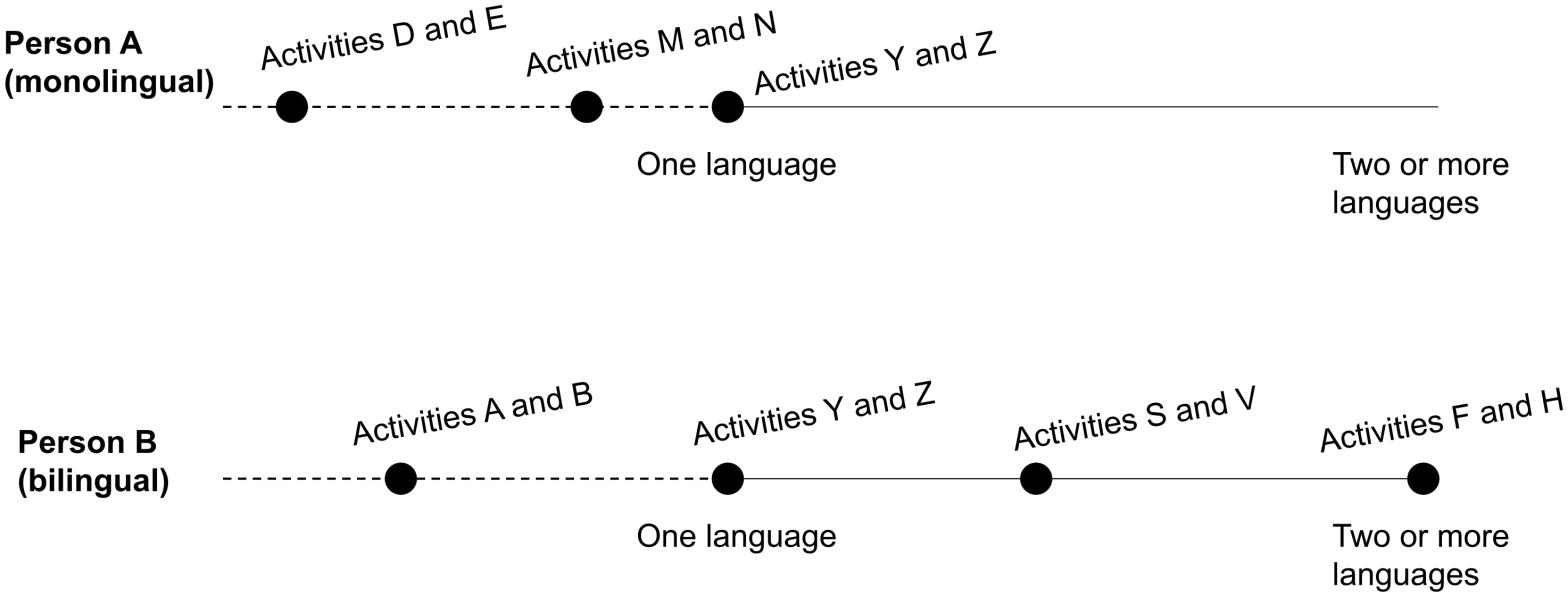
There’s no such thing as literal “full proficiency”: as language skills are grounded in activities, there are always going to be activities for which anyone (whether monolingual or bilingual to some degree) lacks proficiency. To illustrate this, in contrast to the previous figures, here we add space to the left of “One Language” to capture how some activities fall outside the range of an individual’s linguistic repertoire (dotted line).

What Figure 3 still does not capture is the fact that there are activities that an individual is *not* capable of engaging fully successfully in, no matter who the individual is (whether monolingual or bilingual). Consider for instance a single, middle-aged, white monolingual woman who works as the manager of a grocery store in suburban Australia. She will be able to engage in activities such as meeting with supervisors, making schedules, organizing sales events, going bowling with friends, texting with family, reading the local newspaper, booking a flight online, speaking at an Alcoholics Anonymous meeting, and volunteering at a homeless shelter. However, even as a “native” English speaker, this monolingual person may not be able to tactfully fire a difficult employee, negotiate a better salary, read and interpret a complicated medical diagnosis, calm a fearful child, discuss video games with teenagers, interrogate a suspected criminal, deliver a speech at a campaign rally, host a seance, write a legal brief, tell enthralling stories at a fancy dinner party, and so on. Given the uniqueness and unavoidable limitations of each person’s linguistic profile, there are always going to be situations they could not be randomly inserted into and still have sufficient linguistic knowledge to thrive—even in their “native” language. A monolingual person’s particular life experiences allow them to develop language abilities for a wide range of regular activities but still myriad activities will remain outside that person’s scope of ability (see Figure 4).

Once we start thinking of proficiency in terms of activity-grounded skills, it follows that no one is equally proficient as anyone else because no one participates in exactly all and only the same activities as others. In debates surrounding language instruction and testing research, as Shardakova (2022) explains, the view of “proficiency as a single trait (…) was rejected on the grounds of its methodological flaws in the use of statistical analyses and empirical evidence in favor of a complex multifaceted nature of communicative proficiency” (p. 86). Full proficiency is not a single thing, and even so-called “monolinguals” do not possess the linguistic abilities needed to engage equally successfully in *all* of the activities that exist in the society that they live in. Monolinguals vary considerably in language abilities because of the specific contexts in which they develop language skills and are exposed to the languaging practices of others (Dąbrowska, 2012, 2018, 2019; Bice and Kroll, 2019; Gullifer and Titone, 2020; Castro et al., 2022). Describing someone as a “fully proficient bilingual” raises the question: *proficient to succeed in what activities?* And understanding “bilingualism” as monolingual-like proficiency in more than one language does not help, because the question then becomes: *fully proficient like which monolingual?* From these difficulties it follows that “bilingualism” as a category is problematic because it is not *one* thing, just as monolingualism itself is not a single thing: there are as many bilingualisms as there are bilinguals, and there are as many monolingualisms as there are monolinguals—and, as we have been emphasizing from the start, in the real world there are always going to be people in the gray areas where the usual distinctions do not straightforwardly apply.

### “Bilingualism” is problematic because it relies on a problematic distinction between languages

Bilingualism is a problematic category because, in relying on a questionable and unrealistic idea of full proficiency, it also takes for granted a potentially problematic conception of what people are proficient *in*: that is, it presupposes that languages are countable bounded entities. But named languages aren’t objects that exist independently of human activities. As Heller (2007) argues, we need to move away from “a ‘common-sense’, but in fact highly ideologized, view of bilingualism as the coexistence of two linguistic systems” and instead adopt a view of “language as social practice, speakers as social actors and boundaries as products of social action” (p. 1). Recent work coming from many directions motivates rejecting an essentialist view of language by considering the social, historical, and political embeddedness of language practices (see, e.g., Brunstad, 2003; Makoni and Pennycook, 2007; Blommaert, 2010; Lähteenmäki, 2010; Pennycook, 2010; Pennycook and Makoni, 2020; Gullifer and Titone, 2021b; Tiv et al., 2022).

Languages, and particularly standard varieties of languages, are best understood as political instruments (Bourdieu, 1991; Silverstein, 1998/2018). They are powerful markers of group identity, and are used as part of projects to broaden and constrain who is included (Gal and Irvine, 1995). A modern example of a language acting as an umbrella to encompass many different language practices is “Arabic.” For native Arabic speakers, their local vernacular Arabic language is contrasted with “Classical Arabic,” which includes both the historical religious language of the Quran and the Modern Standard Arabic used in formal institutional settings (Haeri, 2003). However, despite Arabic speakers using the same labels to refer to their language practices, it is well documented that the local vernacular varieties of Arabic can vary considerably, posing a challenge to mutual intelligibility (Trentman and Shiri, 2020). The case could be made that in order to speak Arabic to people both in Morocco and in Syria, one has to be bilingual; and yet, a Moroccan and a Syrian would both likely claim that their vernacular is a *variety* of Arabic, rather than a distinct language.^1^ Another example of a standard language which consolidates people across vast territories and spoken varieties is “Mandarin.” In contrast with Modern Standard Arabic, Modern Standard Mandarin was strategically created as part of a larger project of societal reform “in which all the nation’s people would have access to the new official language, and thus increased opportunities for advancement” (Weng, 2018, p. 611). In the cases of both Arabic and Mandarin, there is a powerful single language that groups together what then come to be understood as local variations.

These examples show how named languages can be forces of unification. But they can also act to create divisions, as is the case in Europe for Romance languages (Varvaro, 2013) or Scandinavian languages (Faarlund and Haugen, 2007). Here, languages with significant similarities—e.g., Portuguese and Spanish, or Swedish and Danish—are considered separate languages because of political projects to maintain sovereignty through the promotion of a distinct national identity (Auer, 2005a). Consider the fact that “[s]peakers of Danish, Norwegian, and Swedish normally use their own languages in communicating with one another” (Faarlund and Haugen, 2007). In light of this, can a Swede be considered bilingual if she can use Swedish to communicate effectively with a Dane who is speaking Danish? Or are Swedish and Danish so similar that communication across languages is possible while the speakers remain classified as monolingual? There are also forces within Romance language countries in Europe to create further linguistic distinctions by giving political recognition to languages such as Galician, Catalan, Sardinian, etc. Degrees of differences that in some contexts are seen as merely a distinction between varieties or dialects, in other contexts suffice to distinguish between languages. According to Schneider, “[t]he development of *languages* (and of *dialects*, for that matter) is no socially neutral development but related to political structures and administrative institutions of states, which are co-responsible for the hierarchisation of some varieties into ‘sub’-languages or dialects of others” (Schneider, 2019, p. 4). The dominant variety may be portrayed as objectively superior in terms of grammar, style, etc. when in reality, it is only deemed to be superior because it is the variety of social elites; there is nothing inherently better in the arbitrary variables that distinguish it from “sub-languages.” And by extension, for empirical research, “there is no objective standard for determining when a dialect becomes a language” (Wagner et al., 2022, p. 3).

Although psycholinguistics researchers often acknowledge the complexity of labeling and distinguishing between languages and dialects, the distinctions between and within linguistic varieties are not properly understood unless seen as part of projects that are political in a broad sense, projects of affirming group identity and of differentiation from others (Gal, 2016). While in each context these projects differ in the scope of who’s in and who’s out, they are always about policing some boundary or other. And this poses a fundamental problem for research on “bilingualism.” By definition a person is bilingual because she speaks two (or more) languages. But what counts as a distinct language or merely as a variety of the same language varies widely, and *does not straightforwardly correspond to the amount of linguistic difference people have to be able to navigate*. Some researchers investigate second dialect acquisition as a phenomenon distinct from second language acquisition (e.g., Hazen, 2001; Mordaunt, 2011; Kirk et al., 2014; Wu et al., 2016; Ross and Melinger, 2017; Oschwald et al., 2018), which in turn requires an imperfect decision about how to define dialects as opposed to languages. This decision might be made on the basis of the percentage of lexical similarity (Antoniou et al., 2016) or in the case of Siegel (2010), based not only on several aspects of linguistic similarity and shared history, but also on “the common perception of the speakers of these varieties and not on a technical decision made by linguists” (p. 2). Siegel and others who use this line of reasoning (e.g., Rowe and Grohmann, 2013) do consider the sociolinguistic complexities of the relations between the varieties they study, but the fundamental problem remains that their decision to call some varieties “dialects” is not, and cannot be, established empirically. The trouble remains that what counts as “bilingual” in some contexts requires navigating minimal differences, while in other contexts the obstacles may be more significant and yet not be seen as amounting to a difference between distinct languages, such that the people navigating those obstacles would not technically count as bilingual. Ultimately, there may not be good answers to the question whether bilingualism is cognitively advantageous or disadvantageous because bilingualism is not a single thing.

## What is “cognition” such that something may be cognitively advantageous or disadvantageous?

Is bilingualism cognitively advantageous or disadvantageous? In the previous section we drew from work in sociolinguistics and related fields to identify one type of reason why this research question is problematic: namely, because it is built upon a problematic way of thinking about language(s) and linguistic skills as aspects of human activity in particular contexts. The present section will shift gears to explore a related but different type of reason for seeing the research question as problematic: it relies on an understanding of mind and cognition that’s increasingly disputed by a growing body of research in the sciences of the mind. Before we can try to find out whether bilingualism is cognitively advantageous or not, it’s crucial to examine the assumptions we might be taking for granted concerning the nature of “cognition,” because these assumptions inform how we define “cognitive” advantages and disadvantages in the first place.

Since the “Cognitive Revolution” of the 1950s and 60s, the dominant way of thinking in the sciences of the mind has been to conceptualize “mind” in analogy to computers. In particular, the distinction between software and hardware is often taken to correspond to the distinction between research at two distinct levels of description: these are, on the one hand, research on cognition at the abstract level of the “programs” or “algorithms” underlying mental function and behavior, and on the other hand, research at the level of how our mental software is actually implemented in the brain. The goal of a science of cognition was explicitly articulated along these lines already in its early days (see Neisser, 1967/2014). According to classical cognitivism, then, psychological or mental phenomena are properly explained in terms of two things: *internal knowledge structures* (i.e., symbolic structures that internally represent information about the external world) and *internal mechanisms* for manipulating those knowledge structures (i.e., rules or algorithms for storing the incoming sensory input and processing it, transforming it into some behavioral output).

In this paradigm, the adjective “cognitive” has come to be predominantly used as synonymous with *information processing*. Neisser, one of the pioneers of the computational perspective, articulates quite clearly the intended terminology: “As used here, the term “cognition” refers to all the processes by which the sensory input is transformed, reduced, elaborated, stored, recovered, and used” (Neisser, 1967/2014, p. 4). Understood in this way, cognitive processes are processes of *processing* information. That is, cognition is a process (broadly speaking, a chain of events) in which bits of information are internalized (e.g., some specific type of input derived from sensory stimulation) and then get manipulated (processed, computed) in certain ways that are (hypothesized to be) necessary for supporting the agent’s behavior now or later. Many details in computational approaches to mind have changed over the years, especially following important theoretical and technological advances since the 1970s and 80s (see, e.g., Dreyfus, 1972, 1992; Dennett, 1984; Rumelhart et al., 1986; Fodor and Pylyshyn, 1988); and still, the idea that cognition amounts to information processing remains the “central hypothesis of cognitive science” (Thagard, 2005; see also Pinker, 2005; Gentner, 2019; Thagard, 2019). Accordingly, it’s safe to see this computationalist conception of “cognition” (or something in its vicinity) as the assumption guiding contemporary research on the “cognitive” advantages or disadvantages that bilingualism might have (see, e.g., Grosjean and Li, 2013); in contrast, rejecting the traditional internalist view of cognition and language that focuses on “assessments of individual-level attributes,” and instead acknowledging the inherently social nature of “neurocognitive processes, like language” motivates a “reorienting toward external constraints” (Tiv et al., 2022, p. 13). We will have more to say about this in the last section. For now, in the remainder of this section we consider how work in embodied cognitive science offers a radically different way to understand mind and cognition and, consequently, to approach language and the bilingualism research question.

### Reframing the “cognitive”: Not computational states and procedures, but epistemic relations

A major criticism of classical cognitivism is that, insofar as it equates “cognition” to abstract processes of “information processing,” it thereby also sees cognition as being only marginally related to the body. In this classical view, cognition is informed by the body (i.e., through incoming sensory inputs from the eyes, ears, skin, etc.) and the outcome of cognition is implemented by the body (e.g., through the execution of motor commands in locomotion), but cognition itself is separate and distinct from bodily activity. This construes the body as playing a peripheral role, even in a literal sense, much like the peripherals of computers: these are responsible for the input of information (e.g., mouse, keyboard) and the output of information (e.g., screen, printer), but they are distinct from, and not directly involved in, the computational processing that goes on in between. Hurley (2001) describes this as the “sandwich model” of mind, where cognition is the filling, distinct from the (separate) bread slices of perception and of action: that is, thus understood, “cognition” comprises disembodied, abstract states and processes (the internal mental “cogs”) that are “sandwiched” in between bodily processes of perceptual input and of behavioral output.

All work on “embodied cognition” can be seen as rejecting classical computationalism, but there’s variation between different embodied views when it comes to what exactly is rejected and why. Some work rejects this way of thinking by challenging the *abstractness* inherent to classical computational conceptualizations of cognition. For instance, some researchers propose that the *contents* of cognition are modality-specific because they are grounded in specific bodily experiences, whether sensory (e.g., visual) or motor, or both (Barsalou, 1999; Barsalou et al., 2003). Other researchers have also proposed that the *mechanisms* underlying cognitive function are themselves “embodied” in that neural resources associated with bodily activity also contribute to thought—for instance, when the neural underpinnings of action execution also support imagining that same action (Lakoff and Johnson, 1980a, 1980b; Gallese et al., 1996; Gallese, 1999; Gallese and Lakoff, 2005). These examples illustrate the idea that, rather than cognition being the disembodied processing of disembodied symbolic knowledge structures, the body plays a constitutive role in cognition itself, shaping it both in terms of the representational content (i.e., *what* type of information gets processed) and of the computational procedures (i.e., *how* that information gets processed).

Even while challenging certain aspects of classical computationalism, the versions just mentioned of work on “embodied cognition” clearly leave other aspects unquestioned. On the one hand, they challenge classical views about the nature of the computational states and procedures at play in cognition, namely, questioning their supposed abstractness or disembodied nature, and promoting instead concepts such as “bodily-formatted representation”; but in so doing, on the other hand, they take for granted the computationalist paradigm and, with it, they accept the more fundamental assumption that cognition is properly understood in terms of the storage and processing of bits of information.

**Figure 5:**
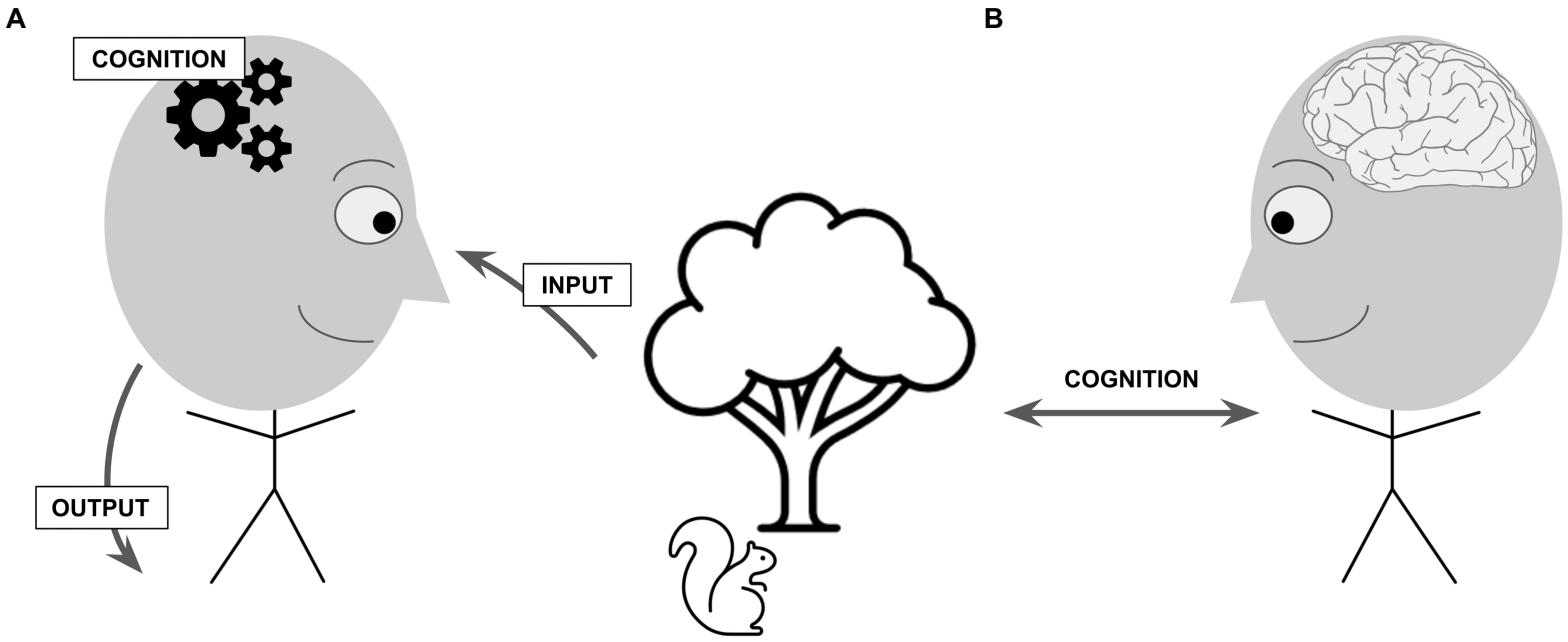
An illustration of competing views of “cognition,” **(A)** understood computationally in terms of representational states and procedures internal to the organism, or **(B)** understood in epistemic terms as a relation of acquaintance or familiarity that holds between an individual and the world. Depending on the particular computational model, the states and processes posited may be understood more or less abstractly, and more or less in connection to neurophysiological descriptions, and so on. In the relational perspective, although the organismic contribution is crucial (including perception-action in all its bodily and neurophysiological dimensions), cognition is not understood to exist “in the head” but rather as a feature of the organism-environment system as a whole.

Our focus here is on a different perspective and line of research in embodied cognitive science, one that rejects these computationalist foundations and that offers an alternative conception of cognition. This radical embodied view is rooted in the functionalist psychology of the end of the 19th century, as developed by the likes of William James and John Dewey, but in some respects it dates even further back (see Green, 1996; Heft, 2001; Crippen and Schulkin, 2020).^2^ In this view, “cognitive” is understood as synonymous not with “computational” (or *that which concerns information processing*) but rather with “epistemic” (or *that which concerns knowledge*). Understood in this way, cognition is still a process, but it’s not a process of *processing* something: it is, rather, a process of *coming to know* something, of becoming familiar with it. Put differently: the computational conception equates cognition to computational states (i.e., bits of information) and procedures (i.e., algorithms for handling those bits of information), which are typically taken to happen inside the organism; in contrast, in the embodied, epistemic conception we see cognition as a *relation* that holds between organism and environment and that, although dependent on organismic processes (e.g., perception-action cycles), is not reducible to what happens inside the organism—cognition is not “in the head,” as Noë (2009) puts it; rather cognition is a feature of the organism-environment system as a whole (see Figure 5).

Let us consider more carefully how exactly the two conceptions differ. Saying that the radical embodied view treats “cognitive” as “having to do with knowledge” might sound like practically the same thing as the computationalist view: after all, the core of the computationalist perspective is the idea that cognition is precisely about what we know and how we store and use that knowledge. So what’s the difference? The key lies in the novelty that the pioneers of cognitive science (in the 1950s/60s onward) introduced for modeling mind and behavior using computing technology and computer-related concepts. This was the idea of operationalizing what someone knows, logically speaking, as statements you could make to express that knowledge, or more technically, as bits of data that encode that information. So, for instance, the fact of your knowing a person (e.g., a friend) or a building (e.g., your friend’s house) came to be operationalized, in light of then emerging computer technology, as internal knowledge structures that represent features of the person or building, from simple facts (e.g., your friend’s hair color, or the number of rooms in the house, which can be assigned a discrete value) all the way up to more complex models (e.g., of what the person looks and sounds like, or the layout of the building). These operationalizations immediately proved that computers could be useful for modeling psychological phenomena, but they quickly came to be interpreted as a theory of what the mind literally is and does.

The computationalist picture is thus built upon the attempt to explain knowledge in terms of what happens inside the organism that knows: this is a view that James criticized, more than a century ago, for holding that “knowledge is explained as the *passage* of something from without into the mind” (James, 1890/1983, p. 215), and in particular that “the mind must in some fashion *contain* what it knows” (p. 472; emphasis added)—or as more recent critics have put it, this is the idea that “one cannot have knowledge of what is *outside* oneself except through the ideas one has *inside* oneself” (Di Paolo et al., 2017, p. 23). The problem is that, as Costall (2007) makes clear, the computationalist picture cannot explain how we gain knowledge through perception because, in that account, “knowledge is invoked to explain perceiving” (p. 22): consider how computational accounts often describe our perception of something as the process of comparing incoming visual inputs to some stored, internal model, based on which we come to categorize the thing now perceived as being X or Y; this description does not explain knowledge because it requires the existence of prior knowledge (i.e., internal knowledge structures) against which incoming sensory inputs are matched—that is, instead of explaining knowledge, it requires that we already have knowledge in the first place.

In contrast, in the radical embodied conception, knowledge (and, by extension, the “cognitive”) is understood, most fundamentally, as a relation rather than a thing.

Generally speaking, relations aren’t reducible to the properties of any one of the things between which the relation holds. Consider, for instance, family relations: you cannot be a cousin or a parent on your own; the roles cannot be reduced to anything about you, because the relation entails the existence of someone else you are related to in that way—put differently, the relation itself exists between the two people rather than inside any one of them. The physical concept of “gravity” provides another example: the fact that a stone, when dropped, falls to the ground is explained not by reference to anything internal and intrinsic to the stone on its own, but rather to the relation between the stone’s mass and the Earth’s, a relation that exists at the interface of the two rather than inside either of them.

Knowledge, then, rather than being an object contained within the mind, is a relation between mind and world: James talks about the “relation of knowing” (James, 1890/1983, p. 212), a relation of “intimacy” or “acquaintance” with some object, a relation that may be more or less articulate—more or less entangled with concepts and language—but whose existence always leads to a transformation in the knower, leaving her changed in her abilities to act in the world as a result of what and how she knows or is familiar with. In the following subsections we draw from two independent but mutually supporting lines of research to further illustrate this embodied, relational epistemic conception of cognition (thereby also illustrating concrete ways to understand the bidirectional arrow shown in Figure 5B).

### Computational “cognitive (dis)advantage” is problematic because it neglects the relational, ecological nature of information

The conceptualization of the “cognitive” in terms of epistemic relations is at the foundation of the research tradition in ecological psychology started by James J. Gibson (Gibson, 1966, 1979; see also, e.g., Richardson et al., 2008; Chemero, 2009, 2013; Turvey, 2018). To illustrate what this relational perspective looks like in more concrete terms, we will consider here Gibson’s claim that “Locomotion and manipulation are neither triggered nor commanded but *controlled,*” to which he added that “they are controlled not by the brain but by information” and, further, that “control lies in the animal-environment system” (Gibson, 1979, p. 225).

Let us flesh out Gibson’s claim. A strictly behaviorist perspective might be to think that locomotion in space and manipulation of some object are elicited (“triggered,” as in Gibson’s quote) from the outside by some stimulus. In contrast, an internalist perspective (e.g., computationalism) would try to explain locomotion and manipulation as caused from within (“commanded,” as Gibson put it), controlled by some motor program. Gibson’s alternative is “ecological” in the same way that ecology as a branch of biological science explains life not at the molecular level (i.e., the level of cells, genes, biochemistry, and so on), but at the “molar” level of the relations between organisms and the environment. Analogously, the explanatory strategy in ecological psychology is to “ask not what’s inside your head, but what your head’s inside of,” as Mace (1977) famously put it: that is, in order to explain an organism’s behavior we need to understand the entire organism-environment system (see, e.g., Reed, 1996; Heft, 2001).^3^

**Figure 6:**
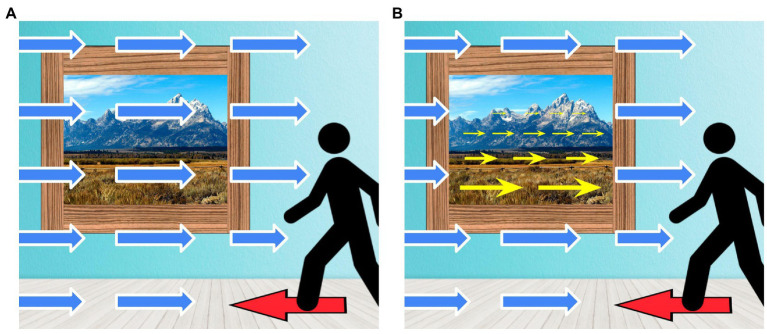
Locomotion generates optic flow, a visual pattern that is directly informative to the agent of her direction of movement. The two images provided here illustrate the optic flow (arrows pointing to the right) that gets generated as a person walks to the left side of the room (red arrow on floor). The specific character of the optic flow can be informative of whether the mountainous landscape is a picture hanging on the wall or an actual scene viewed through a window: if, as you move forward, the optic flow generated is the same everywhere, then what you see is a picture **(A)**, whereas the rate and quality of visual change for the real scene is specific to a space with depth **(B)**. In the computational conception of cognition, locomotion is the *output* of cognition (e.g., the execution of a motor plan) and what you perceive becomes the *input* for further cognitive processing. In contrast, in the ecological view, insofar as it generates optic flow, locomotion is itself cognitive: it is a process through which an agent gains epistemic access to (i.e., comes to know) aspects of the environment and of the agent’s relation to it.

A good example of how, as Gibson put it, locomotion can be controlled *by information* and how control can lie *in the animal-environment system*—rather than controlled by the brain from within the organism—is optic flow (see Figure 6). Optic flow has been described as “the visual streaming or outflow of environmental features that one experiences when moving forward, and inversely, the convergence or inflow of environmental features in the direction from which one is traveling” (Heft, 2001, p. 119). As a pattern of visual displacement, optic flow is a *dynamic* pattern—that is, a pattern of change over time—and it is also an *ecological* pattern—that is, one that emerges from the relation between an organism and the environment as this relation unfolds in space and time. Crucially, optic flow is related to information not because the organism receives and processes bits of information from a supposed visual input: rather, as the organism moves (e.g., by walking) or gets moved (e.g., is carried by others or is transported by a car) the dynamic relational pattern that this movement generates is itself *informative* to the organism of how it is moving with regard to its surroundings, whether forward or backward, for instance. The organism’s task, then, is to *detect* the information (or better, to detect the informative pattern) rather than to internalize and process bits of information. As Bill Warren (2021) puts it in the title of a recent article, “information is where you find it”: that is, “information is available within the constraints of a particular ecological niche” (p. 3) and we just need to *adapt* to it (p. 19). At this point, this idea is no longer speculative, but is a conclusion drawn from decades of research on the role of optic flow in the control of locomotion, yielding ecological explanations of how we steer toward goals and away from obstacles (Warren, 1998; Warren et al., 2001; Fajen and Warren, 2003; Warren, 2006).

Ecological psychology as articulated by Gibson is “a theory of how animals come to *know* their environments—a theory of cognition” (Reed, 1991a, p. 142; see also Reed, 1991b, 1997). In this ecological framing, locomotion controlled by optic flow is a cognitive process in the specific sense that it is a process of coming to know (or coming to be acquainted with) one’s environment and one’s relation to that environment. But it’s not a process of *processing* information: rather, it is one of generating information through movement and detecting information where it is and adapting to it, that is, a process of resonating to informative patterns that arise in the organism-environment relation. The example of locomotion might seem too “bodily” to be relevant for discussions about mind and language, but the point in the ecological approach is precisely that mind and language are always bodily in this way: the goal in the ecological approach is to explain how “mental” things like meaning and the appreciation of meaning are grounded in organism-environment relations as they unfold dynamically through embodied perception-action and as they change through development—see, for instance, ecological views on how people engage with complex and inherently social structures such as the postal system (Gibson, 1979; Heft, 2020).

The ecological way of thinking has implications for a number of debates far beyond the scope of this article. Given our interest in the research question of bilingualism’s cognitive advantages or disadvantages, for present purposes it suffices to indicate how different views of *what* cognition is lead to different interpretations of what the research question is and can be. If we assume cognition to be information processing, then to ask whether or not bilingualism is cognitively advantageous is to ask whether bilingualism contributes to or hinders the processing of information. In contrast, in the radical embodied, ecological perspective “cognitive” is synonymous with “epistemic,” and cognition is understood relationally: as a result, “cognitive” processes are processes at the scale of organism-environment relations through which an agent becomes acquainted with aspects of the environment and those aspects come to guide or steer behavior. From this conception, it follows that to ask whether bilingualism is cognitively advantageous or not is to ask whether bilingualism contributes to or hinders a person’s coming to know the world. Here it might be important to consider individual differences in sensitivity to information (i.e., to the relational informative patterns arising through interaction with the world), but this has very little, if anything, to do with how quickly and efficiently one internalizes and processes bits of information—if that’s even something that people do at all. As this subsection’s title suggests, the computational interpretation of “cognitive” advantages and disadvantages is problematic because it neglects the relational, ecological nature of information: information is not in the head, information does not get processed, and information is never only about the world, but also about the agent’s relation to the world.

### Computational “cognitive (dis)advantage” is problematic because it neglects the relational, situated nature of cognitive processes such as problem solving

Another useful illustration of a relational epistemic view of cognition comes from the distinct but allied tradition of research on “situated cognition” (see, e.g., Suchman, 1987; Lave, 1988; Kirsh, 1991, 2009; Clancey, 1997, 2009; Kirshner and Whitson, 1997, Robbins and Aydede, 2009). To begin, consider how in the classical computationalist perspective it’s assumed that cognitive procedures get implemented in some *context* or other (much like it’s taken for granted that cognition will be implemented by some body or other), yet the cognitive processing itself is thought to be fundamentally neutral with regard to the agent’s situation (much like the processing is supposed to be neutral with regard to the body). In contrast, relational thinking leads to seeing the situation as constitutive of cognition itself: as Clancey (1997) puts it, “every human thought and action is adapted to the environment, that is, *situated*, because what people *perceive*, how they *conceive of their activity*, and what they *physically do* develop together” (pp. 1–2). Here we focus on the implications of this perspective for understanding problem solving.

Since the early days of computational cognitive science, “problem solving” was assumed to be a general cognitive ability or process, perhaps even a foundational one that could explain a wide range of mental and behavioral phenomena (see, e.g., Newell et al., 1958, 1958/1962; Simon and Newell, 1962). But, as Kirsh (2009) points out, “problem solving” cannot be a general cognitive ability or process because “problems” are not general categories independent from particular contexts:

Problems are not regarded to be a distinct category for empirical and computational analysis because what counts as a problem varies from activity to activity. (…) Each problem is tied to a concrete setting and is resolved by reasoning in situation-specific ways, making use of the material and cultural resources locally available. What is called a problem, therefore, depends on the discourse of that activity, and so in a sense, is socially constructed. There is no natural kind called “problem” and no natural kind process called “problem solving” for psychologists to study. Problem solving is merely a form of reasoning that, like all reasoning, is deeply bound up with the activities and context in which it takes place (Kirsh, 2009, pp. 264–265).

In describing cognition as being fundamentally shaped by the situation, Kirsh emphasizes the interactive nature of problem solving. When trying to solve a problem people typically do not first *think* and then *implement* the solution they came up with. Rather, they interact with the elements in their environment throughout the problem solving process, exploring not only possible solutions but even the problem itself: “If it is a word problem (John is half as tall as Mary…), they mutter, they write things down, and they check the question several times. If they are solving an assembly task (here are the parts of a bicycle, assemble it), they will typically feel the pieces, try out trial assemblies, and incrementally work toward a solution” (Kirsh, 2009, pp. 277–278). Through interactive exploration we aren’t simply testing hypotheses about potential solutions, but we are manipulating the environment in ways that test possibilities even before we had contemplated what those possibilities would be. Sometimes we do this by adding structure to the environment, such as when we use physical reminders with sticky notes, or when we rearrange the layout of resources (e.g., books, cooking ingredients, building materials, desktop icons, etc), or when we talk to others as a way to organize our thoughts and actions (Kirsh, 2009, p. 281). Some of these interventions constitute what Kirsh calls “epistemic actions,” as in the example of rotating Tetris pieces as soon as they appear on the screen instead of thinking before implementing a move (see, e.g., Kirsh and Maglio, 1994; Maglio and Kirsh, 1996): these are physical actions that accomplish cognitive work for the agent, revealing and addressing aspects of the situation so that the agent does not have to figure them out reflectively. We solve problems through interaction in and with the world, and what counts as a “problem” and as a “solution” is specific to the situation, which also makes the process of solving a problem inherently situation-specific.

The examples considered so far emphasize the crucial role of what people *do*, in particular circumstances, to provide structure to the problematic situation they are faced with, so as to solve the problem in question. But it’s important to emphasize that, when we do this, we aren’t giving structure to something that lacked structure altogether: rather, we work with the resources and constraints already present and shape them in new ways, but there were resources and constraints already there even before we approached the problem.

**Figure 7:**
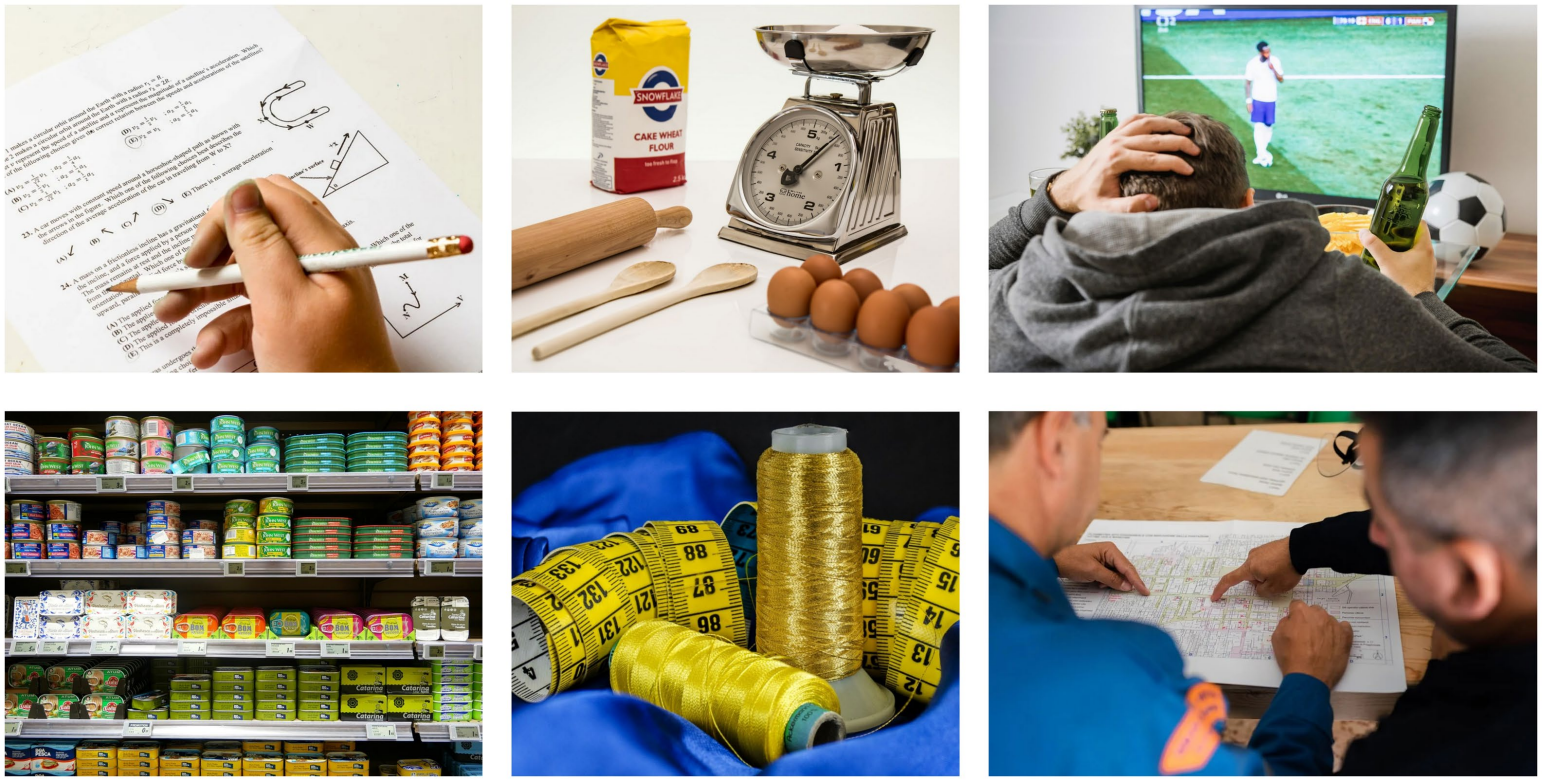
Given the situated nature of cognition, there is no “problem solving in general” because in real life there is no “problem in general” either: problems and solutions are always situation-specific. This is illustrated here with instances in which problem solving is paired to “mathematical reasoning”: rather than a discrete generic ability that merely gets implemented in some context or other, our use of quantity, numbers and their relations always involves local adaptations to situation-specific circumstances that shape the “problem” we are trying to address, for instance at school (top left), when baking (top center), when trying to assess your soccer team’s odds of overcoming an adverse aggregate score where home and away goals are weighted differently (top right), when shopping on a tight budget (bottom left), when sewing (bottom center), and at work as a civil engineer (bottom right).

To illustrate this, consider another classical example from the situated cognition literature—that of going through the checkout line in a supermarket. Anyone who does grocery shopping knows that there are better and worse ways of bagging what you buy: cans and heavy items go first; bread, bananas, tomatoes, eggs and other delicate items go toward the top; but you also need to ensure that bags do not get too heavy, and that the weight is more or less evenly distributed between bags, and so on. As with the previous examples, this kind of scenario has been used to illustrate how cognition is shaped by the ways people act in the environment, exploiting the existing structure, and interactively and iteratively adapting it: as the cashier rings up some items, people commonly use the buffer zone to separate items into distinct categories, to assess how to bag them and in what order (see, e.g., Kirsh, 1995; Solomon, 2007). But to really emphasize the specificity of situations, we think it’s useful to consider a dimension of this example that’s usually left unacknowledged. As we can attest from personal experience, the checkout process at supermarkets in Germany is notoriously fast, surprisingly so for newcomers who have immigrated from North America. Here, the cashier rings up everything as quickly as possible, but in many stores the space past the cash register is minimal, with no buffer zone for sorting and bagging items—instead, there is a separate table or surface nearby where people more calmly organize and pack their groceries after having paid for them. This means that the task here is different: as soon as an item gets scanned, you try to quickly determine where to place it *back in the shopping cart* so that you have an easier time bagging your groceries afterward; of course, you can make your life even easier by organizing items properly on the conveyor belt even before they reach the cashier. The point of this example is that, not only is “problem solving” always a different kind of cognitive process in specific situations (rather than a general process that is merely implemented in different situations), but even a seemingly well-defined situation such as the often-cited “grocery store checkout situation” is not one and the same everywhere—it varies in different contexts depending on what people do and the way the environment is structured (see Figure 7 for additional examples).

So, does bilingualism contribute to or hinder cognition? The upshot of the preceding discussion is that, given the inherently relational, situated nature of cognition, we cannot talk about “cognitive” advantages and disadvantages in general, without accounting for the ways in which cognition in every case is fundamentally shaped by the situation. Here, understanding cognition relationally motivates thinking that cognition is not a single thing, and even problem solving is not a single thing: these are always specific to some situation or other, which also means that they are specific to some agent-environment relation or other. Strategies for coping in some situations can, of course, come to be useful, to varying degrees, for succeeding in other situations. Still, in the perspective outlined here, what counts as success or failure is always going to be situation-specific, such that what counts as an advantage or disadvantage cognitively speaking is not necessarily going to be the same across situations. As suggested by this subsection’s title, the computational interpretation of “cognitive” advantages and disadvantages is problematic because it neglects the relational, situated nature of cognitive processes such as problem solving. Cognitive advantages and disadvantages cannot be reduced to the level of the efficient processing of bits of information supposedly internal to an agent, but must take into account the complexities of how embodied agents relate to resources and constraints in particular real-world situations.

## Discussion

In the introduction we saw how the research question of whether bilingualism is cognitively advantageous or disadvantageous is well established, having guided research at least since the 1960s. But that’s a positive way to think about this. With less of a positive spin, it is interesting to note that, despite all of the developments in research on bilingualism over the years, the progress has not been sufficient to promote a shift in paradigm and we are still guided by a research question as posed more than half a century ago.

In the previous sections we were concerned with sketching how ideas in fields studying language and cognition, and in particular research in sociolinguistics and embodied cognitive science, pose challenges for the usual way of thinking about bilingualism and cognitive advantages or disadvantages. Having done this, we can now more clearly articulate what the research question means given the dominant assumptions about “language” and “cognition.” In the computationalist conception, *cognition* is internal information processing. From this picture of what the mind is and how it works, *language knowledge* comes to be understood as internal representations of linguistic units (e.g., written symbols, sounds) and algorithms or rules about how those units work together (see, e.g., Bucholtz and Hall, 2016). *Bilingualism*, in turn, amounts to the internalization of two or more systems of linguistic units+rules, especially to a level of proficiency in each system equivalent to the proficiency of people who have internalized only one system (i.e., the view of bilingualism as a plurality of monolingualisms within the same person). As a combination of these conceptions, the *research question* can then be interpreted as asking: does having internalized knowledge of more than one system of linguistic units+rules contribute to efficient internal information processing, does it hinder it (i.e., does it pose a burden, for instance, slowing down information processing), or is it neutral in this regard? (see, e.g., Ross and Melinger, 2017; DeLuca et al., 2019, 2020).

In the previous sections we provided a number of different reasons for seeing the research question in this and similar formulations as being deeply problematic and misguided. As we have suggested, ideas from sociolinguistics and embodied cognitive science challenge the essentialist and internalist way of thinking that underlies and motivates the question. But so far we have explored these ideas in separation from one another. Accordingly, our goal in this concluding section is to spell out how the different ideas from the different fields and research traditions come together to motivate a view of cognition, language and linguistic knowledge as embodied, situated, and inherently social.

### Language, cognition, and the social

To make it clearer why—in light of the ideas discussed in the previous sections—the research question of bilingualism’s cognitive advantages and disadvantages is problematic, consider the following related but different question:

Is being “lingual” cognitively advantageous, disadvantageous or neutral? That is, is having some linguistic knowledge and ability (as opposed to lacking any and all linguistic knowledge and ability) a cognitive boon?

Although it might sound far-fetched, this new question could be applied to the case of a feral child raised by non-human animals in the wild: in comparison, is a person who grows up in human environments and develops some communication skills better off—and not only better off socially, for instance, or in terms of well-being, or chance of surviving to old age, but better off *cognitively*?

Would this question be a reasonable and fruitful starting point for research, one that would lead to decades of work trying to find evidence of advantage, disadvantage, or neutrality? It does not seem like it. Our intuition is that this is not a great research question because the answer is obvious: regardless of how you conceptualize “language” and “cognition,” having some linguistic communicative ability is, for humans, always cognitively better than not having any. But why is that? We think the ideas from sociolinguistics and embodied cognitive science reviewed in the previous sections provide some useful guidance. Those ideas give us a way to make sense of *what we come to know when we learn language*, and *what it means to know it*—or, put differently, they give us a particular conceptualization of the nature and object of linguistic knowledge. In clarifying what we mean by this, we will be building upon Jean Lave’s view of learning as not “a process of socially shared cognition that results in the end in the internalization of knowledge by individuals, but as a process of becoming a member of a sustained community of practice” (Lave, 1991, p. 65). This is a claim Lave makes about learning in general that we think is especially helpful for thinking about language.

First, *what we come to know when we learn language*: we come to know the *world*—though not in the abstract and in general, but in the concrete way in which it is experienced in some community of practice or other. In particular, when we learn language we come to know a way of relating to things, to people, and to events—some of which are named, categorized, described and evaluated, referred to in marked and unmarked ways, and ultimately (or, in the first place) noticed, while others aren’t. Learning language, we propose, is developing a way of *being* in the world: for Martin Heidegger (1927/2001) being and world are inseparable because being human is always “being-in-the-world”; and as Merleau-Ponty (1945) emphasizes, “being-in-the-world” is inherently social, interpersonal, relational (see also, e.g., Gallagher, 2012; Käufer and Chemero, 2021).

Following from this, and second, *what it means to know language*: it means to have skill in *participating* in the relevant community of practice. This includes, for instance, having skill in coordinating with others and contributing to shared activities, but it’s not limited to interpersonal action. Even when we are acting individually and in supposed isolation from others, we cannot help but do so in certain ways that are informed by, and recognizable in light of, a socially-shared frame of reference. In the limit case, even the actions of a hermit who fled civilization as an adult would likely continue to be *intelligible* to a hypothetical observer from the society the hermit fled, however deplorable the hermit’s actions might be—a radically unintelligible action, by contrast, could not be deplorable.

Skill in participating in a community of practice can look different in different communities of practice. It often involves participation in particular practices of codifying behavior, including, in some cases, ability to engage in explicit meta-linguistic talk about correct and incorrect ways of speaking and writing, and about why X is right but Y is wrong, and so on. An intuitive example of this phenomenon concerns developing skill in navigating expectations around dominant, prestige varieties in a given language—for instance, coming to be able to discuss the correct use of standard expressions or the pronunciation of technical terms within a particular disciplinary academic circle in Standard American English. But the same can be present in any community of practice: practices of codifying behavior and of discussing those codifications can arise around anything that gets used to form social groups, be it around profession, religious membership, racial identity, and so on.

Still, meta-linguistic skill is arguably *not* necessary for linguistic skill. This is a point in which the relational embodied perspective we are sketching differs from the traditional essentialist and internalist perspective in which linguistic knowledge is precisely the internalization of meta-linguistic rules. In our view, it’s possible to succeed in participating in a community of (communicative) practice without being able to articulate the patterns that make up participation in the community, that is, without being able to put your finger on precisely why this is right and that is wrong in the way of speaking, for instance. Developing the ability to engage in meta-linguistic coordination (especially when this is already part of the community of practice) is a case of expansion in linguistic skill rather than acquisition of a separate, distinct, and supposedly more fundamental, skill. And crucially, like all other expansions of linguistic skill (including, e.g., “learning a new language”), developing the ability to engage in meta-linguistic coordination is *necessarily cognitively advantageous*. This is because expansions in linguistic skill always broaden the scope of what and how we can know: new aspects of the world are unveiled and can be confronted and can come to be understood and made sense of, resulting in activity that is more sensitive to the particularities of the situation. Recognition of the centrality of the social in language through participation in communities of practice calls for further consideration of how power and political interests come into play.

### Language, social construction, and power

In the section titled “What is ‘bilingualism’ such that it may be cognitively advantageous or disadvantageous?” we talked about how the boundaries between languages and within languages are used politically as a means of creating division as well as uniting people groups. But it’s not just that languages exist out there and are sometimes used politically. The existence of languages as distinct bounded entities was not the origin of their political use: rather, their coming to be conceptualized as distinct bounded entities is an outcome of their (social, political) history.

Named languages are socially constructed in more than one sense. First, they are socially constructed in that terms like “Spanish,” “Russian,” “Indonesian,” and “Quechua” do not capture natural kinds, but reflect a convention to name a complex set of practices (and not others) as a single thing. Many of these names are the legacy of historical processes of European nation building (Blommaert and Verschueren, 1992; Irvine et al., 2009) and of European colonialism (Makoni and Pennycook, 2007). So, “English” as an idea or concept exists because people made it up. This does not entail, however, that named languages are *not real*. Socially-constructed or made-up things are very real: e.g., money, weeks, national borders, etc.

In another sense, named languages are socially constructed in that, at specific points in time and space, people in interaction with one another developed shared communicative behaviors that solidified certain patterns that they came to identify as *their* language, even if they may not have used the term “language” to refer to those practices or to separate them from other practices in their culture. Put another way, languages came to be conceptualized as distinct bounded entities, and through this, they came to *be* distinct bounded entities. Blackledge and Creese (2014) caution that, “[t]he idea of ‘a language’ therefore may be important as a social construct, but it is not suited as an analytical lens through which to view language practice” (p. 1). As socially constructed in the ways just identified, named languages are still real, and understanding the socially-constructed categorization schemes that people are subjected to as speakers of single or multiple named languages can still help shed light on real phenomena.

Discrimination is not a bug, but a feature.

If we view bilingualism only in additive and cognitive terms (speaking more than one language brings benefits), we miss the point that bilingualism is more usefully understood in terms of “resources which circulate in unequal ways in social networks and discursive spaces” (Pennycook, 2019, p. 169, quoting Heller, 2007, p. 2).

A discussion of bilingualism would not be minimally adequate without acknowledging the role of language status and speaker status to support prejudice and justify processes of social stratification (Piller, 2015). Woolard (2020) affirms that beliefs about languages “endow some linguistic features or varieties with greater value than others, for some circumstances and some speakers” (p. 2). Not all language practices are treated equally, and double standards abound, such that bilingualism is inconsistently and unequally defined and valued (Piller, 2012). To illustrate, consider the case of two people who could be labeled bilingual in Spanish and English: an Anglo-American teenager who studies Spanish in school and a Mexican-American peer who is proficient in English and Spanish as a result of bi-cultural life experiences. While the white English speaker is praised for acquiring valuable (“marketable”) additional language skills, the Mexican-American student faces linguistic discrimination and is denied access to a host of opportunities. Bilingualism even in the same languages will not be treated equally if the speakers’ status is not equal (Piller, 2012). This means that being bilingual will confer different advantages and disadvantages to different people, even *cognitive* advantages and disadvantages—being denied access to experiences limits possibilities to learn and come to know the world, which is a kind of epistemic deprivation.

The example of the two teenagers connects to Politzer’s (1981) description of the influence of class difference on bilingualism: “Within the upper ranges of socioeconomic status, bilingualism tends to be associated with some additional educational advantages; within the lower ranges, it often appears to result in an additional handicap” (p. 3). Social factors such as race (see, e.g., Rosa and Flores, 2017), class (see, e.g., Block, 2013), and immigration status (see, e.g., Piller, 2001) are central in understanding how value is unequally accorded to (bilingual) language practices. And this same double standard in bilingualism applies within monolingual contexts too. Not all monolinguals are created equal, which is clear, for instance, in cases of discrimination against the English of various racialized people groups in the US (see, e.g., Milroy and Milroy, 1985/2012; Lippi-Green, 2012). As Blommaert (2001) points out, “people can be ‘majority’ members (e.g., they can speak the language of the ruling groups in society) yet they can be thoroughly disenfranchised because of a lack of access to status *varieties* of the so-called ‘power-language’” (p. 136, emphasis added). All language practices are connected to power, and unequal treatment of language users occurs both for those considered bilingual and for those considered monolingual. Classification of people according to their language practices is not neutral and cannot be properly understood apart from processes of social differentiation for the maintenance of hierarchy.

The idea that languages are discrete, natural objects that are used by distinct speech communities is common, and perhaps still the dominant understanding of language today. Under this ideology, even victims of linguistic discrimination might recognize that the discrimination they suffer is of sociopolitical basis, but might still not realize that the criterion used in this particular form of discrimination (i.e., language) is itself of sociopolitical origin as well. That is, while acknowledging the arbitrariness of the fact of discrimination, they might still assume languages to be real, natural, and objective categories rather than socially-constructed objects that are inherently instrumentalized precisely in the service of social discrimination.

In line with this, and as we suggested previously, rather than uncritically embracing essentialism about bounded languages (e.g., thinking that there is such a thing as “the English language”), it is better to think of languages as goals, projects. This applies to large dominant languages, such as in the promotion of Swahili as a regional *lingua franca* (Amidu, 1995); it also applies to projects in minority language revitalization, such as, for instance, in the teaching of the Irish language in schools in Ireland (Ceallaigh and Dhonnabhain, 2015). Whether majority or minority, languages are aspirational, ideal (Costa, 2020). Languages are what some people want the world to be like (which includes human activity, and aspects of interpersonal relations). From this perspective, “language”—as a collective pattern of coordination and joint activity—is only properly understood *relationally*: this is clear, for instance, in the way standard varieties can only be defined in contrast with what are considered nonstandard varieties (Auer, 2005b); accordingly, attempts to distinguish between varieties are always a move to solidify difference, and through this, to “realize” (i.e., “make real”) a sociopolitical state of affairs.

An analogous point applies to “language” at the personal level too. With regard to real languaging practices, rather than focusing on the named languages that people speak, it is perhaps better to think in terms of idiolects, namely unique assemblages of bits of language that make up the linguistic repertoire of an individual speaker (Blommaert, 2009). Individuals speak idiolects that work in some contexts, for some ends, because they at least partially overlap with the idiolects of others participating in those contexts and activities. So people who can speak “English” participate in a complex pattern of multiple partly-overlapping idiolects. As such, relational thinking also applies to “language” understood at the personal level: after all, a person’s languaging is what it is because of, and in interaction with, others—and this includes many different “others,” that is, both “others” who are seen as interlocutors and co-participants, on the one hand, and, on the other hand, those ‘radically other’ others, who aren’t interlocutors and co-participants, not even remotely.

Relational thinking thus motivates shifting the focus away from rigid classifications of people and people groups solely on the basis of named languages and of categories like “bilingual” and “monolingual”: instead, the relational nature of language at both the collective and personal levels calls for careful attention to how both individuals and groups forge their unique linguistic profile in specific contexts and activities. This involves taking seriously how both individual repertoires and collective patterns of interpersonal overlaps are defined relationally, through participation in communities of practices, and distinction between communities of practice. And it also involves taking seriously the relational *cognitive* dimension of these practices: we cannot properly make sense of what cognitive advantages or disadvantages bilingualism might provide without considering how opportunities for learning and growing in our knowledge of the world (including contributing to shared knowledge production) are mediated by language and by the way linguistic ability is interwoven with particular practices, group membership and sociopolitical projects of world-making.

### What now?

The ideas examined in the previous sections motivate a radical departure from usual interpretations of the research question of whether bilingualism is cognitively advantageous or not. On the one hand, as we have seen “bilingualism” is a complicated category, and describing someone as “bilingual” is not as straightforward as ascribing a trait such as “being X centimeters tall,” but is instead more like describing them as “tall enough to play this game” or “too tall to stand up here” or “too short to be able to see us from there”—in other words, it’s a relational feature, a characteristic not of people on their own but of the different ways people can relate to their environment and participate in different activities. This emphasis on the multifaceted nature of bilingualism is in line with the work mentioned earlier that takes into account multiple, dynamically changing variables to define the potentially very different linguistic profiles of different bilinguals (e.g., Hartanto and Yang, 2016, 2020; Gullifer et al., 2018; DeLuca et al., 2019, 2020; Gullifer and Titone, 2020, 2021a; Sulpizio et al., 2020; Kremin and Byers-Heinlein, 2021). By blurring the boundaries between “bilingual” and “monolingual,” these multivariate accounts are also promising starting points for understanding any and all linguistic profiles, including those of people typically described as monolinguals but who may differ widely from other monolinguals in their range of experiences and skills. On the other hand, however, even these more sophisticated and fine-grained multivariate measures of bilingualism aren’t enough to support conclusions about the cognitive benefits or drawbacks of bilingualism without further consideration of what “cognitive” advantages and disadvantages are in the first place.

As traditionally construed, the cognitive advantage/disadvantage question is a question about whether knowledge of multiple languages (understood as internalized representations of multiple linguistic systems or codes) enhances the efficiency of, or poses a burden to, information processing. But work in radical embodied cognitive science like that explored earlier in the paper challenges this view of cognition as information processing, instead understanding the “cognitive” in terms of an epistemic relation constituted by embodied, situated interaction. In this view, as we saw, even walking is a “cognitive” process, yet this is not because it involves information processing, but rather because it’s an epistemically enriching act—by moving around and exploring the environment agents can change how and what they know, for instance, revealing or occluding different aspects of the environment (as illustrated in Figure 6).

So, consider how, as cited earlier in the paper, bilingualism is often linked to enhanced executive control and executive function as shown in tasks involving self-monitoring and the inhibition of irrelevant information, as well as slower lexical access and retrieval (e.g., Bialystok et al., 2008; Festman et al., 2010; Pelham and Abrams, 2014). In line with traditional computational thinking, these effects are often considered “cognitive” because they are construed as changes in information processing speed and accuracy as managed by a “master subsystem, the central executive” that “controls and coordinates the resources of the cognitive system” (Anastas et al., 2014, p. 263). Here, reaction time or response time is a common measure of information load and processing efficiency: whatever imposes a “cognitive” burden tends to lead to a slowing down of performance, because “the executive could only operate as quickly as the slowest component” that it gets inputs from (Anastas et al., 2014, p. 268; see also, e.g., West, 2001; Diamond, 2013; Karbach and Unger, 2014; Vasquez et al., 2018). Although common, this way to construe “executive function” is neither theoretically-neutral nor unproblematic. As some researchers in embodied cognitive science have put it, the “deeply rooted acceptance that behavior’s organization reflects entirely internal, locally defined, representational processes has made the dependence of standard theory on executive function so pervasive as to be almost invisible” (Richardson et al., 2008, p. 163). By rejecting such internalist, localist and computationalist assumptions about cognition, authors like these also challenge the usual understanding of what the label “executive function” is supposed to capture. For instance, some have proposed that, rather than amounting to the output of an internal master component that oversees and *organizes* the flow of data, the phenomena falling under the label of “executive function” are instead *self-organized*, emerging dynamically through the interaction of system activity at different timescales (Anastas et al., 2014; Kelty-Stephen et al., 2016). The implication of views like these is that “executive function” is not cognitive because it entails information processing (in particular, a process of “managing” and “organizing” data flow), but rather because, understood properly, it is an epistemic enrichment of embodied, situated perception-action. If the “cognitive” is not operationalized in terms of the internal storage and processing of information but is instead understood in terms of the behavioral implications of what and how one knows, then speed is no longer obviously a crucial factor. If after learning something you come to know to slow down and pay closer attention to a given task you are confronted with, then this is a cognitive boon compared to lacking this sensitivity to the situation and proceeding quickly as you might have done before. Sometimes knowing more (or differently) means taking your time to do something that demands more attention or precision. Taking longer is not necessarily a cognitive deficit (nor symptomatic of a cognitive deficit), and sometimes it might even be cognitively (i.e., epistemically) advantageous. Ultimately, then, a shift in how we understand not only “bilingualism” but also “cognition” has to be taken into account if we are to make sense of how speaking two or more languages has cognitive implications for embodied, situated agents—that is, implications for what and how they know.

These points suggest that the research question we are focusing on in this paper is weird in that it rests on strange assumptions about human communication and our mental lives, assumptions that neglect the situated and embodied nature of our experience. But the research question is also “WEIRD” in that it is characteristic of a way of thinking typical of “Western, Educated, Industrialized, Rich and Democratic” societies (Henrich et al., 2010). Monolingualism is not the norm in most of the world today nor has it been the norm in most of human history. And yet, monolingualism is the presumed reality in the most influential centers of knowledge production today, such that studies tend to take bilingualism as an exotic phenomenon to be explained, while monolingualism is the default for normal people (this is evidenced by the common reference to “bilingualism” as synonymous with knowledge of “additional” languages—a description that erroneously presupposes monolingualism to be the normal starting point upon which something may be added or not). Because of this monolingual bias in WEIRD science, although Figures 1–4 were not meant to depict a temporal relation between monolingualism and bilingualism, we would not be surprised if some readers saw those figures as illustrating a chronological progression where you start out as a monolingual and later move in the direction of learning additional languages. Of course, the reality is that there are plenty of individuals in many places today and throughout history for whom monolingualism is not the starting point (see, e.g., Grosjean, 2010 for a nuanced look at the experiences of contemporary bilinguals, and Canagarajah and Liyanage, 2012 for a discussion of multilingual practices in the pre-colonial southern hemisphere). Citing Doerr’s (2009) work, Lovrits and de Bres (2021) assert that “the ideological prerequisite of innate monolingualism in a standard language exerts a strong influence on constructions of linguistic legitimacy and competence” (p. 400). Along these lines, some psycholinguists acknowledge the danger of taking for granted, and reinforcing, the “dominant ideology of monolingualism as a gold standard” (Tiv et al., 2021): given its prevalence globally, it’s inadequate to treat bilingualism as the exceptional case (Gullifer and Titone, 2021b). And in a provocative hypothetical scenario, Pennycook and Makoni (2020) suggest that, if the dominant centers of knowledge production today were located in the Global South, bilingualism (rather than monolingualism) would be assumed to be the norm, and researchers might be interested in understanding the rare, exotic peoples who are so limited in their communicative abilities that they can only speak a single language—though perhaps these researchers would not even think of languages as countable entities analyzable in separation from other aspects of human life.

Given these reflections and how they cast doubt on the research question of bilingualism’s cognitive advantages or disadvantages, we want to conclude with a few suggestions of potentially helpful guides for future research.

A first practical and foundational implication of the preceding discussion is that we need to be extremely careful in experimental design and how we delineate the target population and choose participants. The ways we see some people as monolingual, bilingual, “native” speakers, and so on are permeated with philosophically-loaded assumptions. It’s crucial that researchers address these assumptions head on: rather than uncritically importing the procedures and groupings others have used previously, results cannot properly count as findings if they do not specify which assumptions the research presupposes.

Second, ideas like the ones explored in this paper motivate moving away from research that takes for granted *named languages* as if they are the only natural way, or even a necessary way, of partitioning phenomena of linguistic communication. Once we get over the bias toward named languages and move toward partitioning linguistic communication around *activities*, a related shift makes sense, away from focusing on speech communities and toward focusing on communities of practice, which encompass patterns of speech and communication, but where those are only understood as grounded in the real-world activities of, and relations between, co-practitioners.

Third, besides moving away from partitioning communication around named languages, the ideas explored in this paper also support the related but distinct shift away from a focus on languages as countable entities (many of which would supposedly coexist inside a person) toward the study of an individual’s idiolect (or total languaging repertoire) as a social, embodied meaning-making practice. As in the previous point, this fits nicely with attention to activities and communities of practice, as it is in these that “languaging” gets molded through behaviors of codification and interpersonal coordination. But the move away from languages as countable entities also invites re-consideration of how we understand all that individuals are capable of, how we qualify and quantify their skill, and the ways in which people are more or less well equipped for thriving in different circumstances. Instead of asking how many languages someone can speak, a better question is what they can get done, and what they can participate in, and in what contexts.

Here we think researchers interested in bilingualism would have much to profit from theoretical and methodological developments in recent work on languaging practices in embodied cognitive science. This includes studies of language through the lens of embodied interactivity (see, e.g., Steffensen, 2017; Steffensen and Harvey, 2018; Li et al., 2020), studies focusing on the tuning of multimodal embodied interaction over developmental timescales (see, e.g., Rączaszek-Leonardi et al., 2013, 2018; Nomikou et al., 2016; Rohlfing et al., 2019), as well as studies using nonlinear analysis methods to quantify the dynamics of coupling and complexity matching in interpersonal coordination (see, e.g., Richardson and Dale, 2005; Abney et al., 2014; Coey et al., 2016). Examples such as these tend to focus on embodied dimensions of interaction in only one language, but, even in light of the caveats above, we see promise in using similar conceptual frameworks and methodological tools for investigating diverse multilingual practices while taking seriously the complex embodied and relational nature of languaging.

Lastly, we think it’s important to understand what this different way of thinking of language and cognition means for elucidating the role that technology can play in human embodied activity. “In the wild” people use whatever resources are available to them in order to be able to get by. Is using one’s phone to translate something necessarily a crutch, something external and distinct from the individual’s language, language knowledge and cognition “themselves”? Views of cognition as distributed and extended and as a feature of the organism-environment relation motivate moving away from these conclusions. The smartphone in this case can be seen as a resource that is both linguistic and cognitive, a resource that supports and partly constitutes the person’s ability to coordinate with others or to solve a problem and through this to achieve cognitive ends, making sense of the world and continuing moving forward more successfully than before. This example illustrates the more general upshot: understanding language requires understanding what resources people rely on, when, where, and why, and what their use leads to.

## Data availability statement

The original contributions presented in the study are included in the article/supplementary material, further inquiries can be directed to the corresponding author.

## Author contributions

All authors listed have made a substantial, direct and intellectual contribution to the work, and approved it for publication.

## Funding

We acknowledge support by the German Research Foundation and the Open Access Publication Fund of TU Berlin.

## Conflict of interest

The authors declare that the research was conducted in the absence of any commercial or financial relationships that could be construed as a potential conflict of interest.

## Publisher’s Note

All claims expressed in this article are solely those of the authors and do not necessarily represent those of their affiliated organizations, or those of the publisher, the editors and the reviewers. Any product that may be evaluated in this article, or claim that may be made by its manufacturer, is not guaranteed or endorsed by the publisher.

## Footnotes

^1^Anecdotally, a Franco-Syrian colleague fluent in both French and Arabic told us that it took her a month of immersion to understand Arabic spoken in Morocco, the same amount of immersion time she needed to be able to understand Italian based on her knowledge of French. From this experience, the description of one contrast as holding between dialects and the other as between separate languages would appear to be artificial.

^2^Awareness of this history should inform how we see the "burden of proof" in contemporary research. That is, some see the radical embodied conception as a recent invention that needs to be justified and shown to be superior to the computationalist conception it’s trying to replace. But it’s historically more accurate to see it as an older idea potentially worth rescuing, and in particular one that the computationalist conception itself can be evaluated as a new competitor to.

^3^To be sure, approaching psychological phenomena through an ecological lens does not amount to "blackboxing" the brain, but it does call for rethinking the way we understand how the brain works and, in particular, how what the brain does contributes to the organism-environment system (see, e.g., Van Orden et al., 2012; Dotov 2014; Bruineberg and Rietveld 2019).
